# Rapid onset of anaemia in a patient with alcoholic cirrhosis: The clue might be in the smear

**DOI:** 10.1002/jha2.320

**Published:** 2021-10-20

**Authors:** Sander De Bruyne, Simon Degandt, Timothy Ghys

**Affiliations:** ^1^ Department of Laboratory Medicine AZ Sint‐Lucas Ghent Belgium

A 64‐year‐old woman with a medical history of alcoholic cirrhosis (diagnosed 7 months prior), monoclonal gammopathy, and hemochromatosis presented to the emergency department with abdominal discomfort, general weakness and loss of appetite. On admission, laboratory analysis revealed a mild macrocytic anaemia (haemoglobin 10.2 g/dL), high total bilirubin (4.9 mg/dL), and normal lactate dehydrogenase (193 U/L) levels. During the consecutive days, an ongoing decline in haemoglobin levels (nadir on day 7: 7.4 g/dL) was noted in parallel with a rise in reticulocyte counts (day 7: 38/10^3^ RBC), total bilirubin (day 7: 9.3 mg/dL) and lactate dehydrogenase (day 7: 329 U/L) levels. Subsequently, the patient received two units of packed red blood cells, which only resulted in a slight and short‐lasting improvement of haemoglobin levels. While a direct Coombs test appeared to be positive, the result was interpreted as being false positive due to the presence of interfering paraproteins (IgG‐kappa type). However, peripheral blood smear analysis unravelled marked poikilocytosis with the presence of numerous spur cells (also called acanthocytes). Spur cells are erythrocytes that acquire spinous projections on their surface due to the occurrence of alterations in the lipid metabolism of cirrhosis patients. The accumulation of excess cholesterol in the cell membrane gives rise to a reduction of deformability, thereby making them prone to haemolysis. Figure 1A illustrates the blood smears on days 1, 3, and 6 of the hospitalization period. A clear increase in spur cells from 2% to 87% of the erythrocytes can be observed over the course of time. Moreover, Figure 1B shows a relationship between increasing percentages of spur cells, decreasing levels of haemoglobin (solid blue line, reference ranges are depicted with dashed blue lines), and increasing levels of total bilirubin (solid orange line, reference ranges are depicted with dashed orange lines). The prognosis of spur‐cell anaemia in cirrhosis patients is known to be poor and the patient deceased 9 days after admission due to the consequences of a septic shock.

**FIGURE 1:**
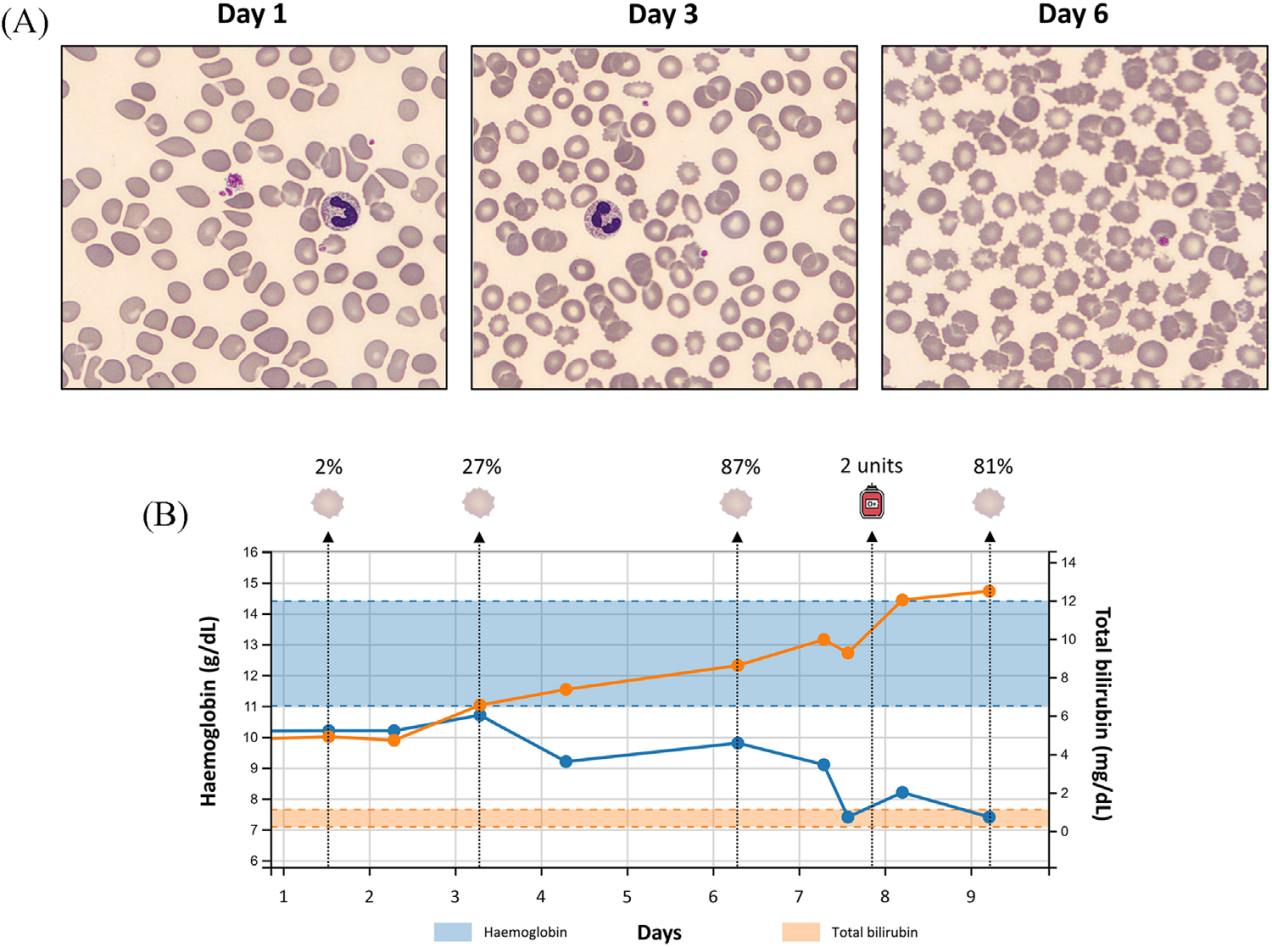
(A) Blood smears on day 1, 3, and 6 of the hospitalization period showing a clear increase in spur cells. (B) Relationship between increasing percentages of spur cells, decreasing levels of haemoglobin (solid blue line, reference ranges are depicted with dashed blue lines), and increasing levels of total bilirubin (solid orange line, reference ranges are depicted with dashed orange lines)

## CONFLICT OF INTEREST

The authors declare no conflict of interest.

## Data Availability

Data sharing not applicable to this article as no datasets were generated or analysed during the current study.

